# Ablation-First Balloon-Assisted Acetabuloplasty for Painful Acetabular Metastases: Clinical Outcomes and Mechanical Rationale

**DOI:** 10.3390/curroncol33040217

**Published:** 2026-04-15

**Authors:** Claudio Pusceddu, Eliodoro Faiella, Pierluigi Maria Rinaldi, Jesús Ares-Vidal, José Maria Maiques Llacér, Igor Radalov, Albert Solano López, Salvatore Marsico

**Affiliations:** 1 Radiology, Mater Olbia Hospital, SS 125 Orientale Sarda, 07026 Olbia, Italy; 2 Diagnostica per Immagini, Fondazione Policlinico Universitario Campus Bio-Medico, Via Álvaro del Portillo 200, 00128 Rome, Italy; 3 Department of Radiology, Hospital Del Mar, Pg. Marítim de la Barceloneta 25-29, Ciutat Vella, 08003 Barcelona, Spain

**Keywords:** acetabular metastases, balloon-assisted acetabuloplasty, thermal ablation, radiofrequency ablation, microwave ablation, cementoplasty, interventional oncology

## Abstract

Purpose: To evaluate the safety, technical feasibility, and clinical outcomes of a strict two-step protocol—CT/fluoroscopic-guided thermal ablation followed by Balloon-Assisted Acetabuloplasty (BAA)—for the treatment of painful acetabular metastases, utilized as an immediate mechanical stabilization bridge prior to radiotherapy. Materials and Methods: A retrospective study was conducted on 16 consecutive patients treated for severe mechanical pain (VAS ≥ 6) and impaired mobility due to osteolytic acetabular metastases. The physiological rationale mandated a strict procedural sequence: (1) preliminary thermal devitalization using radiofrequency or microwave ablation to reduce tumor pressure and vascularity, followed subsequently by (2) balloon-assisted cavity compaction and polymethylmethacrylate (PMMA) cement injection. Clinical outcomes included VAS for pain and the Functional Mobility Scale (FMS) assessed before treatment and up to 6 months post-procedure. Results: Technical success was 100% with a mean procedural time of 58 ± 14 min. No major complications occurred. At a mean follow-up of 8.2 months, all 16 patients were alive. The procedure yielded dramatic acute pain relief: mean baseline VAS dropped from 7.4 ± 0.8 to 2.3 ± 1.0 at 1 week, and to 0.9 ± 0.9 at 1 month (*p* < 0.001), remaining stable at 6 months. Functional mobility was rapidly restored, with mean FMS improving from 2.9 ± 0.7 pre-procedure to 1.1 ± 0.3 at 1 month (*p* < 0.001), allowing independent ambulation in 87.5% of patients. Conclusion: The strict “ablation-first” BAA strategy is safe and highly effective. It abolishes load-bearing pain and restores biomechanical stability immediately, allowing previously immobilized patients to rapidly regain independent ambulation and seamlessly transition to necessary consolidative radiotherapy.

## Simple Summary

Cancer spreading to the hip bone causes severe pain and makes walking incredibly difficult. While standard cement injections offer a minimally invasive alternative to risky open surgeries, controlling the liquid cement in large bone defects remains challenging. This study demonstrates a highly effective solution using a strict, two-step sequence: first, utilizing percutaneous heat ablation to shrink the tumor and directly reduce pain, and then using a balloon to ensure the stabilizing cement is injected with a controlled distribution and adequate volume to firmly stabilize the acetabulum. As a result, patients experience immediate pain relief and can rapidly regain their ability to walk independently. This reliable technique will impact real-world oncology by allowing previously immobilized patients to recover quickly and comfortably transition to necessary radiation treatments.

## 1. Introduction

Balloon kyphoplasty (BKP) is a well-established minimally invasive procedure for the treatment of vertebral compression fractures, initially developed for osteoporotic and neoplastic lesions [1,2]. The technique involves the percutaneous placement of cannulas into the vertebral body through which inflatable balloons are inserted. Balloon inflation allows partial restoration of vertebral height, compaction of trabecular bone, and the creation of a cavity for subsequent low-pressure injection of bone cement (polymethylmethacrylate, PMMA) after balloon removal [1,3,4].

The application of these biomechanical principles has recently been extended beyond the spine. Metastatic osteolytic lesions of the periacetabular region represent a significant clinical challenge in oncologic patients. Due to the massive weight-bearing forces exerted on the pelvis, these lesions frequently cause severe mechanical pain, structural instability, impending fractures, impaired ambulation, and a drastic reduction in quality of life [5,6,7]. The periacetabular region is a common site for skeletal metastases, particularly originating from breast, renal, and prostate carcinomas, as well as multiple myeloma [6,8].

Traditional management of acetabular metastases relies on a multimodal approach, including systemic chemotherapy, osteoclast inhibitors (bisphosphonates, denosumab), analgesics, and local external beam radiotherapy [6]. When conservative treatments fail or when an impending fracture is present, surgical reconstruction is often required. However, open acetabular reconstruction procedures, such as the modified Harrington technique combined with total hip arthroplasty, are associated with high perioperative complication rates (30–36%), significant blood loss, prolonged hospitalization, and undesirable interruptions of systemic oncologic therapies [5,7,9].

Percutaneous acetabuloplasty has emerged as a minimally invasive alternative, applying cement augmentation principles directly to the pelvic region [6,8,10]. While clinical studies have demonstrated that standard percutaneous acetabuloplasty provides significant pain relief [6,8,11,12], controlling the extravasation of liquid cement in large, anatomically complex, and highly vascularized osteolytic pelvic defects remains a substantial technical limitation. Balloon-Assisted Acetabuloplasty (BAA) aims to resolve this issue by creating a defined cavity. However, the optimal sequencing of the interventional steps—specifically regarding when to perform tumor ablation—remains heavily debated in current practice.

Therefore, the rationale of this study is to evaluate a rigorous clinical pathway: the procedure must strictly commence first with thermal ablation for rapid tumor cytoreduction, desensitization, and intraosseous pressure decompression, followed only subsequently by balloon-assisted cementoplasty. Specifically, this study aims to report the clinical outcomes of this specific sequence when intentionally utilized to immediately restore structural stability and facilitate the subsequent delivery of radiotherapy, addressing a crucial gap in the literature regarding the exact sequencing of local interventions to optimize patient recovery.

## 2. Materials and Methods

### 2.1. Study Design and Patient Population

A retrospective observational study was performed on 16 consecutive patients treated between May 2019 and February 2025. The study received institutional review board approval, and all patients provided informed consent.

Patient Selection and Multidisciplinary Tumor Board (MTB) Evaluation: Patient selection was strictly governed by a Multidisciplinary Tumor Board (MTB). For all 16 patients, the MTB explicitly decided to alter the traditional treatment sequence, which typically favors upfront radiotherapy. Because the patients presented with severe mechanical pain (VAS ≥ 6) and significant weight-bearing impairment due to substantial osteolysis in the load-bearing dome of the acetabulum, the MTB consensus was to perform the “ablation-first BAA” protocol prior to radiation.

Inclusion Criteria: (i) Histologically confirmed malignancy with acetabular metastasis; (ii) Severe mechanical pain (VAS ≥ 6); (iii) Impaired functional mobility (FMS ≥ 2); (iv) Preserved cortical containment sufficient for cement injection; (v) Life expectancy ≥ 3 months.

### 2.2. Pre-Procedural Assessment and Systemic Therapies

Patients underwent meticulous clinical and imaging evaluation (contrast-enhanced CT or MRI) to define lesion geometry and proximity to neurovascular structures (e.g., sciatic nerve). Pain intensity was measured using the Visual Analog Scale (VAS, 0–10). Functional mobility was assessed using the Functional Mobility Scale (FMS): 1 (Independent), 2 (Ambulatory with aids), 3 (Wheelchair), and 4 (Bedridden). To ensure methodological consistency and to accurately isolate the acute biomechanical stabilization provided by the procedure from the delayed effects of subsequent radiotherapy, FMS scores were systematically recorded for all patients at two precise and standardized timepoints: at baseline (pre-procedure) and exactly at 1 month post-procedure. All patients were actively receiving systemic oncological therapies appropriate for their primary malignancies. Furthermore, to manage generalized skeletal-related events, a subset of the cohort was actively receiving bone-modifying agents at standard oncological doses: 3 patients were treated with zoledronic acid (4 mg intravenously every 3–4 weeks) and 2 patients were treated with denosumab (120 mg subcutaneously every 4 weeks). Preoperative antibiotic prophylaxis (cefazolin 2 g) was administered 30 min prior to the intervention.

### 2.3. Technique: The Strict BAA Protocol

Procedures were performed under dual CT and fluoroscopic guidance. Patients were positioned in lateral decubitus under conscious sedation (continuous intravenous fentanyl citrate infusion) and local periosteal anesthesia.

Step 1—Thermal Ablation (The Obligatory First Step): Crucially, mechanical manipulation of the metastasis was strictly prohibited until thermal devitalization was achieved. Performing ablation first induces rapid coagulative necrosis, drastically reducing tumor vascularity, halting bleeding, and lowering the inherently high intraosseous pressure.

**Figure 1:**
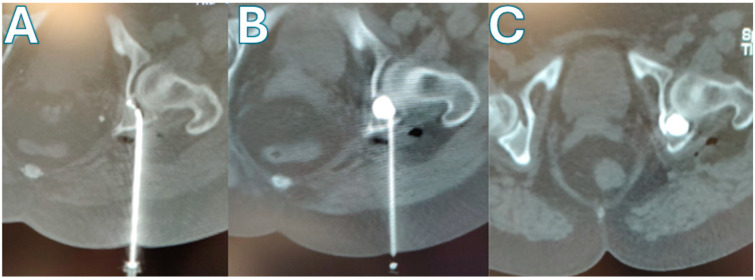
CT/fluoroscopic-guided Balloon-Assisted Acetabuloplasty in a 53-year-old patient with metastatic breast carcinoma involving the left posterior acetabulum. (**A**) Axial CT image showing percutaneous placement of a curved radiofrequency ablation probe within the osteolytic acetabular lesion. (**B**) Balloon-assisted cavity creation using a curved balloon catheter; contrast opacification confirms adequate expansion and cavity formation within the ablated lesion. (**C**) Post-procedural CT demonstrating satisfactory PMMA cement filling of the lesion with appropriate distribution and no evidence of intra-articular leakage.

**Figure 2:**
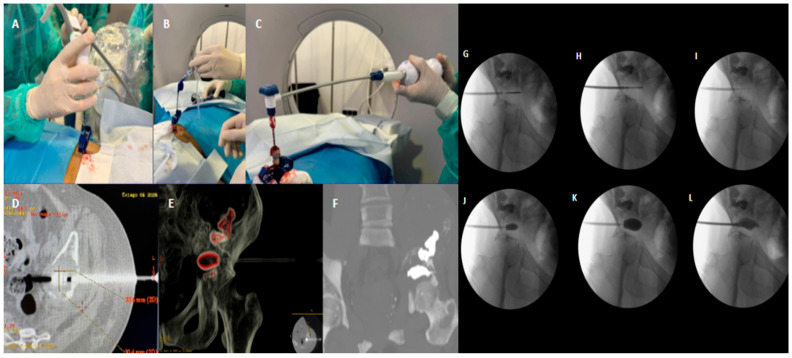
CT/fluoroscopic-guided thermal ablation followed by Balloon-Assisted Acetabuloplasty in a 47-year-old female patient with lung carcinoma and a painful metastatic lesion of the left acetabulum. (**A**) Sterile preparation of the operative field and initial percutaneous setup at the planned entry site. (**B**) Fluoroscopic-guided advancement of the coaxial bone access system toward the osteolytic lesion within the acetabular roof, along a safe trajectory to the weight-bearing dome. (**C**) Insertion of the straight microwave ablation antenna through the coaxial access system into the target lesion. (**D**) Axial CT image confirming accurate intralesional positioning of the microwave ablation antenna within the osteolytic metastatic focus of the acetabulum. (**E**) Three-dimensional CT reconstruction depicting lesion morphology and its spatial relationship with the acetabular roof, useful for procedural planning and confirmation of the treatment corridor. (**F**) Post-procedural CT image demonstrating satisfactory intralesional distribution of polymethylmethacrylate (PMMA) cement within the treated acetabular cavity, with effective structural reinforcement of the load-bearing region. (**G**) Fluoroscopic image confirming correct positioning of the microwave ablation antenna within the lesion before completion of the ablation phase. (**H**) Fluoroscopic image showing creation of the intralesional cavity using a drill after thermal ablation, in order to prepare an adequate space for subsequent balloon insertion and controlled expansion. (**I**) Introduction of the balloon catheter in its deflated state into the previously prepared cavity. (**J**) Initial inflation of the balloon with contrast medium under continuous fluoroscopic monitoring, allowing progressive cavity expansion and compaction of the devitalized tumor tissue. (**K**) Full balloon expansion with contrast, obtaining a well-defined cavity within the acetabular dome suitable for controlled cement filling. (**L**) Final cementation phase under real-time fluoroscopic guidance, showing PMMA injection into the created cavity with satisfactory filling of the lesion and final mechanical stabilization.

**Figure 3:**
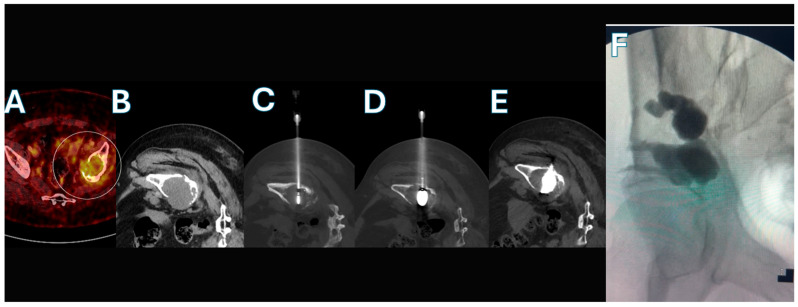
CT/fluoroscopic-guided thermal ablation followed by Balloon-Assisted Acetabuloplasty in a 42-year-old male patient with pancreatic carcinoma and a painful osteolytic metastatic lesion of the right acetabulum. (**A**) Axial PET-CT image demonstrating intense FDG uptake within the osteolytic metastatic lesion of the right acetabulum (circled). (**B**) Axial non-contrast CT confirming the presence of a large osteolytic lesion involving the weight-bearing dome of the right acetabulum. (**C**) Axial CT image showing positioning of the microwave ablation antenna centrally within the lesion during the ablation phase. (**D**) Axial CT image demonstrating balloon catheter inflation within the ablated cavity, allowing controlled expansion and compaction of devitalized tumor tissue. (**E**) Axial CT image after balloon deflation showing the created cavity filled with polymethylmethacrylate (PMMA) cement, achieving structural stabilization of the acetabulum. (**F**) Final fluoroscopic anteroposterior (AP) view confirming adequate cement distribution within the acetabulum without significant extravasation.

• Group A (Steerable System, *n* = 12): Utilized a Steerable Radiofrequency Ablation System (STAR™, Merit Medical Systems, Inc., South Jordan, UT, USA) with articulating probes to navigate eccentric geometries within the acetabular roof (Figure 1).
• Group B (Straight System, *n* = 4): Utilized Microwave Ablation (AMICA-GEN, HS Hospital Service S.p.A., Aprilia, Italy) for lesions with a direct, linear access path (Figure 2 and Figure 3).

Step 2—Balloon-Assisted Cavity Creation and Cementation: Following adequate thermal ablation, an inflatable balloon (iVAS™, Stryker, Kalamazoo, MI, USA, or Arcadia™, Merit Medical Systems, Inc., South Jordan, UT, USA, depending on the ablation system used) was introduced coaxially into the ablated necrotic tract. The balloon was slowly inflated under CT guidance to mechanically compact the devitalized tissue against the surrounding bone, creating a distinct void. After balloon deflation and removal, ultra-high-viscosity PMMA was injected under continuous real-time fluoroscopic monitoring. A non-enhanced CT scan was performed immediately post-procedure to evaluate the final PMMA distribution.

### 2.4. Post-Procedural Radiotherapy Integration

Following recovery from the BAA procedure (typically within 2–3 weeks), all patients were referred back to the radiation oncology department to undergo their previously planned external beam radiotherapy to consolidate local disease control.

### 2.5. Statistical Analysis

Changes in VAS and FMS over time (pre-op, 1 week, 1 month, 3 months, 6 months) were assessed using the Wilcoxon signed-rank test. A *p*-value of <0.05 was considered statistically significant.

## 3. Results (Table 1)

### 3.1. Demographics and Procedural Characteristics

The cohort comprised 16 patients (9 females, 7 males) with a mean age of 63.4 ± 9.4 years. The primary malignancies were breast cancer (*n* = 5, 31%), lung cancer (*n* = 4, 25%), renal cell carcinoma (*n* = 4, 25%), pancreatic cancer (*n* = 2, 12.5%), and oral cavity squamous cell carcinoma (*n* = 1, 6.5%).

Technical success—defined as the successful completion of both the ablation and cementation steps with adequate lesion filling—was achieved in all 16 cases (100%). The overall mean duration of the procedure was 58 ± 14 min. At the time of data analysis, at a mean follow-up of 8.2 months (range 6–12 months), all 16 patients were alive.

No major complications occurred. A minor, asymptomatic linear cement leak along the needle track was observed in only 1 case (6.2%), requiring no intervention.

**Table 1:** Summary of Patient Demographics, Procedural Characteristics, and Clinical Outcomes.

Parameter	Value
Patient Demographics	
Total number of patients	16
Gender (Female/Male)	9 (56.3%)/7 (43.7%)
Mean Age (years) ± SD	63.4 ± 9.4 (Range: 44–77)
Primary Malignancies	
Breast	5 (31.3%)
Lung	4 (25.0%)
Kidney (Renal Cell Carcinoma)	4 (25.0%)
Pancreas	2 (12.5%)
Oral Cavity	1 (6.2%)
Procedural Characteristics	
Technical Success	16 (100%)
Group A (Curved RF + Steerable Balloon)	12 (75%)
Group B (Straight MWA + Straight Balloon)	4 (25%)
Mean Procedure Time (minutes) ± SD	58 ± 14
Major Complications	0 (0%)
Minor Complications (Asymptomatic Leak)	1 (6.2%)
Post-Procedural Radiotherapy Completion	16 (100%)
Clinical Outcomes (VAS)	Mean ± SD (Median)
Pre-operative VAS	7.4 ± 0.8 (7.0)
1 Week Post-Op VAS	2.3 ± 1.0 (2.0) *
1 Month Post-Op VAS	0.9 ± 0.9 (0.5) *
3 Months Post-Op VAS	0.4 ± 0.6 (0.0) *
6 Months Post-Op VAS	0.4 ± 0.6 (0.0) *
Functional Mobility Scale (FMS)	Mean ± SD (Median)
Pre-operative FMS	2.9 ± 0.7 (3.0)
1 Month Post-Op FMS	1.1 ± 0.3 (1.0) *

* *p* < 0.001 compared to baseline.

### 3.2. Clinical Outcomes: Acute and Sustained Pain Relief (VAS)

The “ablation-first” BAA procedure resulted in profound and immediate pain relief.

• Pre-operative VAS: Mean 7.4 ± 0.8 (Median: 7)
• 1 Week Post-Op: Mean 2.3 ± 1.0 (*p* < 0.001 vs. baseline)
• 1 Month Post-Op: Mean 0.9 ± 0.9 (*p* < 0.001)
• 3 Months Post-Op: Mean 0.4 ± 0.6
• 6 Months Post-Op: Sustained at 0.4 ± 0.6

Systemic analgesic drug therapy (NSAIDs and opioids) was significantly reduced or completely discontinued just 1 week after treatment in 13 out of 16 patients (81%).

### 3.3. Functional Recovery (FMS)

The immediate restoration of mechanical stability translated into rapid restoration of ambulation.

• Pre-operative FMS: Mean 2.9 ± 0.7. Most patients were severely limited or wheelchair-bound, with two patients being completely bedridden (FMS 4).
• At the standardized 1-month follow-up: Mean 1.1 ± 0.3 (*p* < 0.001). The improvement was dramatic; 14 out of 16 patients (87.5%) regained normal, independent ambulation.

### 3.4. Seamless Radiotherapy Integration

The multidisciplinary strategy was flawlessly executed. All 16 patients (100%) successfully underwent their subsequently scheduled radiotherapy. The rapid analgesia provided by the BAA procedure allowed patients to tolerate the hard, flat table of the RT linear accelerator without pain, optimizing targeting and delivery.

## 4. Discussion

Osteolytic acetabular metastases frequently present as oncologic emergencies due to disabling mechanical pain and rapid functional decline [5,6,7]. The primary finding of this study is that executing a rigorous physiological sequence—CT/fluoroscopic-guided thermal ablation performed strictly as the primary step, followed by Balloon-Assisted Acetabuloplasty (BAA)—is highly safe and exceptionally effective. In our cohort, this sequence yielded a 100% technical success rate, drastically reduced pain (mean VAS from 7.4 to 0.9 at one month), and restored independent ambulation in 87.5% of patients, successfully acting as an immediate biomechanical bridge to consolidative radiotherapy.

This study presents several limitations that must be carefully considered when interpreting the results. First, this is a retrospective analysis involving a relatively small cohort (*n* = 16) from a highly selected patient population. The reader should be aware that this small sample size limits the statistical power of our findings and increases the susceptibility to selection bias. Consequently, while we report a 100% technical success rate and an absence of major complications, these safety metrics must be interpreted with caution, as rare adverse events may only become apparent in larger series. Second, the absence of a control group (e.g., patients treated solely with radiotherapy, conventional cementoplasty, or open surgery) precludes any claims of definitive comparative superiority. Therefore, while our data strongly support the acute efficacy and safety of the “ablation-first” BAA protocol, the conclusions drawn should be viewed as robust observational evidence rather than definitive proof that this technique outperforms other established modalities.

Third, regarding functional outcomes, our evaluation of ambulation (FMS) was strictly limited to a single, standardized 1-month postoperative timepoint. While this timing was intentionally designed to isolate the immediate mechanical benefits of the intervention prior to the onset of radiobiological responses, it fundamentally restricts our conclusions. Because functional capacity naturally fluctuates over time due to systemic disease progression, skeletal-related events in other districts, or general oncologic decline, the reader cannot extrapolate this acute restoration of independence to long-term functional survival. Finally, while the 8.2-month overall follow-up is sufficient to capture acute pain relief and short-term structural stability, it is inadequate for assessing late mechanical failures, long-term implant durability, or the delayed risk of secondary fractures in patients with extended survival.

A critical analytical question is whether the dramatic clinical improvement observed can be definitively attributed to the BAA procedure itself, given that all patients subsequently underwent radiotherapy and received concurrent systemic oncological treatments (including bisphosphonates or denosumab in a subset of patients). While long-term local tumor control is undeniably the synergistic result of a multimodal approach, the acute symptomatic and functional responses must be exclusively attributed to the interventional procedure [13]. The pharmacology of bone-modifying agents and the radiobiology of external beam radiotherapy operate on a delayed timeline. These treatments inhibit future osteoclast activity and halt progressive osteolysis over a period of weeks to months; however, they do not reverse existing macroscopic osteolytic cavities or provide any immediate structural support [6]. The acetabulum is subject to massive mechanical weight-bearing loads. The severe baseline pain in our cohort (VAS 7.4) was primarily mechanical nociceptive pain driven by micro-instability and impending cortical collapse. Therefore, the immediate, precipitous drop in VAS scores recorded at just 1 week (from 7.4 to 2.3) and the rapid restoration of ambulation cannot be biologically attributed to RT or medical therapies. As corroborated by recent literature evaluating the biomechanics of the osseous pelvis and consolidative treatments [13], this acute relief is the direct consequence of immediate biomechanical stabilization—distributing axial loads through the PMMA cement—combined with the acute desensitization of the highly innervated periosteum achieved by the preliminary thermal ablation.

Balloon-assisted acetabuloplasty represents a logical biomechanical evolution of percutaneous cementoplasty. Conventional percutaneous acetabuloplasty provides meaningful pain relief, but its main technical limitation remains the unpredictability of cement distribution in large irregular osteolytic cavities. The use of inflatable balloons addresses this limitation by allowing the creation of a controlled cavity within the lesion. Kurup et al. described this technique in a cohort of seven patients treated with cryoablation followed by balloon-assisted osteoplasty, demonstrating excellent PMMA distribution with a median tumor filling rate of 63% and minimal cement leakage [14].

The procedural sequence itself likely contributed substantially to the excellent safety profile observed in our study (only 6% asymptomatic leakage). In our protocol, thermal ablation was considered the obligatory first step. Ablation induces coagulative necrosis, reduces tumor vascularity, limits intralesional bleeding, and lowers the elevated intraosseous pressure typical of metastatic lesions. Only after this step was the balloon introduced. This ablation-first rationale aligns closely with recent multimodal treatment strategies known as AORIF (Ablation, Osteoplasty, Reinforcement, Internal Fixation) [7,15]. Dussik et al. reported excellent results using the AORIF technique in 50 patients, observing significant improvements in pain and ambulation scores (*p* < 0.001) and enabling 19 out of 22 initially non-ambulatory patients to walk again [15]. Biomechanical studies further validate these controlled augmentation techniques; Lea et al. demonstrated that combining percutaneous interventions with cement augmentation provides the highest mechanical load resistance (4711 ± 362 N), representing a 12% increase compared to cement alone and a 125% increase compared to screw fixation alone [16].

Other emerging minimally invasive techniques include photodynamic balloon stabilization using the IlluminOss system [17,18]. Prospective studies by Lozano-Calderon et al. involving 30–39 patients demonstrated significant pain reduction (VAS decreasing from 60–80 preoperatively to 0–30 at six weeks) and a substantial reduction in opioid use from 100% to 20% at six months using this technology [17,18].

When comparing these percutaneous approaches to open surgical reconstruction, the advantages of minimally invasive techniques become particularly evident in the advanced oncologic setting. Colman et al. compared 11 patients treated with percutaneous acetabuloplasty to 17 patients undergoing open reconstruction for Harrington grade II–III lesions [19]. While the surgical group demonstrated slightly greater pain reduction at three months, percutaneous techniques offer crucial advantages: reduced morbidity, minimal blood loss, significantly shorter hospitalization, and the ability to continue systemic oncologic therapies without prolonged interruption [6,15,20,21]. Furthermore, percutaneous approaches allow rapid functional recovery and preserve the native bone stock, leaving open the possibility of conversion to a total hip arthroplasty (THA) if it becomes necessary later [5,20,22]. Even when compared to modern open reconstructions using porous tantalum implants [23,24], percutaneous cement augmentation offers a less invasive alternative with a significantly lower perioperative complication profile. Complications associated with balloon-assisted acetabuloplasty are generally rare and minor; major adverse events such as septic arthritis, symptomatic nerve compression, or the need for surgical conversion have been reported only rarely in the literature [8,11,14,21], which is consistent with our own experience of zero major complications.

Table 2 provides a comprehensive overview of recent key studies evaluating percutaneous acetabular stabilization techniques.

Evaluating local disease control following BAA combined with RT presents specific imaging challenges. PMMA cement introduces significant beam-hardening artifacts on standard CT and magnetic susceptibility artifacts on MRI, potentially masking local recurrences. However, the utilization of advanced imaging techniques successfully mitigates these issues. Dual-energy CT (DECT) with monochromatic reconstructions significantly reduces beam-hardening, and Metal Artifact Reduction Sequences (MARS) on MRI allow for adequate surveillance [14,21]. Reassuringly, utilizing these modalities, no evidence of local tumor progression or mechanical failure was noted in our cohort during the follow-up period.

From a technical and guidance perspective, while combined CT and fluoroscopy remains the absolute gold standard for navigating deep intraosseous pelvic anatomy safely, recent literature highlights an emerging role for Ultrasound (US) in musculoskeletal oncology interventions [25]. While US cannot penetrate intact cortical bone to guide the deep cementation steps, it serves as an invaluable, radiation-free adjunct for pre-procedural assessment and initial access. Specifically, for lesions that exhibit extensive cortical destruction or possess a significant soft-tissue component, US can effectively guide initial core-needle biopsies, evaluate tumor vascularity via color Doppler, and dynamically map the trajectory to avoid critical adjacent neurovascular structures (such as the sciatic nerve or femoral vessels) prior to cortical penetration [25].

Finally, it is essential to contextualize this approach against the paradigm of treating with Radiotherapy (RT) alone. While RT alone remains the standard of care for painful, uncomplicated bone metastases, it is insufficient as a standalone acute treatment for lesions involving impending fracture or severe mechanical instability of the acetabulum. RT fails to provide the necessary acute structural support, leaving the patient at high risk of catastrophic joint collapse during the weeks required for re-ossification [6]. By strictly adhering to the “ablation-first BAA” protocol, we bridge this critical temporal gap. The procedure immediately stabilizes the joint, abolishes load-bearing agony, and allows the patient to comfortably tolerate the rigid positioning required for subsequent RT sessions.

## 5. Conclusions

Balloon-Assisted Acetabuloplasty (BAA) is a highly effective, minimally invasive surgical technique for the management of debilitating acetabular metastases. The data clearly demonstrate that executing the procedure with a strict physiological rationale—performing tumor ablation first to reduce pressure and bleeding, followed by balloon compaction and cementation—guarantees exceptional procedural safety, minimal leakage, and optimal structural reinforcement.

Furthermore, this study highlights the critical value of a multidisciplinary approach. By electing to perform BAA prior to radiotherapy, we achieved an immediate and dramatic reversal of functional disability. This stabilized, pain-free state created the ideal clinical conditions for the subsequent delivery of consolidative radiotherapy, cementing BAA as a powerful first-line intervention in the oncologic treatment paradigm for complex, unstable pelvic lesions.

## Figures and Tables

**Table 2:** Summary of selected studies on percutaneous stabilization techniques for acetabular metastases, including pain relief, functional outcomes, and complication rates.

Study	Year	Technique	N	Pain Outcome	Functional Outcome	Key Findings	Complications
Kurup AN et al. [14]	2015	Cryoablation + BAA	7	Significant reduction (VAS ↓)	Improved ambulation	Median tumor filling 63%, controlled cement distribution	29% asymptomatic cement leakage
Hartung MP et al. [21]	2016	Ablation + Cement + Screws	15	Significant VAS reduction	Improved mobility	Combined stabilization increases mechanical resistance	Minor leaks, no major complications
Durfee RA et al. [6]	2017	Percutaneous acetabuloplasty	12	Moderate pain reduction	Partial functional recovery	Minimally invasive alternative to surgery	No major complications
Dussik CM et al. [15]	2023	AORIF (Ablation + Cement + Fixation)	50	VAS ↓ (*p* < 0.001)	86% regained ambulation	Multimodal percutaneous approach highly effective	Minor complications only
Lozano-Calderon SA et al. [17]	2024	Photodynamic nail stabilization	30	VAS 60 → 30	Reduced opioid use	Innovative intramedullary stabilization	Low complication rate
Lozano-Calderon SA et al. [18]	2024	Photodynamic nail stabilization	39	VAS 80 → 0–30	Functional improvement	Sustained pain control at 6 months	Minimal complications
Colman MW et al. [19]	2015	Percutaneous vs. Open surgery	28	Greater reduction with surgery	Longer recovery with surgery	Percutaneous = lower morbidity	Higher complications in surgery group
Lea WB et al. [16]	2021	Biomechanical study (cement ± fixation)	Experimental	—	—	Cement + fixation ↑ load resistance (up to +125%)	Not applicable
Current Study	2026	Ablation-first + BAA	16	VAS 7.4 → 0.9 (1 month)	87.5% independent ambulation	Immediate stabilization + bridge to RT	6% asymptomatic leakage

## Data Availability

The original contributions presented in this study are included in the article. Further inquiries can be directed to the corresponding author.
